# A cell differentiation landscape for monocyte and interstitial macrophage in the lung with diffuse alveolar damage

**DOI:** 10.1093/procel/pwaf070

**Published:** 2025-08-04

**Authors:** Duo Su, Mengyun Deng, Lingfei Hu, Hao Xie, Bo Yang, Huiying Yang, Dongsheng Zhou

**Affiliations:** State Key Laboratory of Pathogen and Biosecurity, Academy of Military Medical Sciences, Beijing 100071, China; Reproductive Genetics Center, Bethune International Peace Hospital, Shijiazhuang 050082, China; State Key Laboratory of Pathogen and Biosecurity, Academy of Military Medical Sciences, Beijing 100071, China; State Key Laboratory of Pathogen and Biosecurity, Academy of Military Medical Sciences, Beijing 100071, China; State Key Laboratory of Pathogen and Biosecurity, Academy of Military Medical Sciences, Beijing 100071, China; Reproductive Genetics Center, Bethune International Peace Hospital, Shijiazhuang 050082, China; State Key Laboratory of Pathogen and Biosecurity, Academy of Military Medical Sciences, Beijing 100071, China; State Key Laboratory of Pathogen and Biosecurity, Academy of Military Medical Sciences, Beijing 100071, China

**Keywords:** diffuse alveolar damage, immunology, interstitial macrophage, monocyte, GDF15, single-cell transcriptomics

## Abstract

Diffuse alveolar damage (DAD) is recognized as a deadly type of acute inflammatory lung injury caused by toxic inhalants, but its cellular and molecular pathogenesis remains largely unclear. In this study, by using a mouse model of ricin-induced DAD, we explored the heterogeneity of recruited monocyte (Mono) and Mono-derived interstitial macrophage (IM) in the DAD lung. There was the development of 2 distinct IM subsets, namely IM^pi^ (pro-inflammatory) and IM^ai^ (anti-inflammatory), from recruited Mono^pi^. A subset of recruited Mono^pi^ could get the proliferating phenotype (namely pMono^pi^), and meanwhile pMono^pi^ served as the intermediate of Mono^pi^-to-IM^ai^ transition. The presence of growth differentiation factor 15 (GDF15) facilitated Mono^pi^-to-pMono^pi^-to-IM^ai^ transition, whereas GDF15 deficiency exerted the negative feedback effect of enhancing Mono^pi^-to-IM^pi^ shift. These findings provided a cell differentiation landscape for Mono and IM in the DAD lung, which would promote a deeper understanding of cellular immunology of DAD and offer a theoretical basis for developing novel therapeutic strategies against acute lung injury.

## Introduction

Ricin is an abundant protein component of castor beans and represents one of the most potent poisons known in the world (Abbes et al., 2021). Ricin poses considerable risks to public health because of its wide environmental persistence and extreme toxicity (Bolt et al., 2023). The main mode of ricin intoxication is accidental or misused ingestion of castor seeds, which are freely and widely available (Abbes et al., 2021). When ricin is made into a purified material, exposure can occur through the air, food, or water (Schep et al., 2009). The use of ricin for terror attacks has been documented (Janik et al., 2019), and ricin is categorized by the US Centers for Disease Control and Prevention as a category B bioterrorism agent (CDC, 2000). Ricin is a disulfide-linked glycoprotein composed of an enzymatic A chain and a cell-binding B chain, and it can inhibit protein synthesis by depurinating a specific adenosine from 28S rRNA and thus arresting mRNA translation (Lord et al., 2003).

During inflammation, there are 2 functionally distinct Mono subsets recruited to inflammatory tissues such as the lung from the blood, and they can be distinguished by their surface protein Ly6C and chemokine receptors CCR2 and CX3CR1: (i) pro-inflammatory Mono (Mono^pi^, CD11b^+^Ly6C^hi^CCR2^hi^CX3CR1^lo^) recruited through CCL2-CCR2 axis, and (ii) anti-inflammatory Mono (Mono^ai^, CD11b^+^Ly6C^lo^CCR2^lo^CX3CR1^hi^) through CX3CL1/CX3CR1 axis (Guilliams et al., 2018). During acute lung inflammation, Mono^pi^ can transition into Mono^ai^ and also engraft to generate Mono-derived interstitial macrophage (IM) (Moore et al., 2023; Teh et al., 2022; Vanneste et al., 2023). Typical mature Mono is non-proliferating, but Mono can reenter the cell cycle to acquire the proliferating phenotype to further undergo Mono-to-IM transition in the inflammatory lung tissues (Moore et al., 2023; Teh et al., 2022; Vanneste et al., 2023). Mono-derived IM can be divided into the pro-inflammatory IM (IM^pi^, CD11c^hi^ Ly6C^hi^CX3CR1^lo^), often referred to as “classically activated” M1 population, and the anti-inflammatory IM (IM^ai^, CD11c^lo^Ly6C^lo^CX3CR1^hi^), broadly described as “alternatively activated” M2 population (Moore et al., 2023). Although studies have explored the dynamics of Mono-to-IM transition during acute lung inflammation (Moore et al., 2023; Teh et al., 2022; Vanneste et al., 2023), the developmental pathways and the molecular regulation of this process remain largely unexplored.

Diffuse alveolar damage (DAD) is a deadly type of acute inflammatory lung injury caused by toxic inhalants (Cardinal-Fernandez et al., 2017). The toxicity of ricin varies based on the routes of exposure, with pulmonary inhalation posing the highest risk (Audi et al., 2005; Stoll et al., 2023). As shown in our previous studies (Deng et al., 2022; Jiao et al., 2021; Su et al., 2023), aerosolized intratracheal inoculation of the lethal doses of ricin in mice could lead to DAD, which was characteristic of significant recruitment of monocyte (Mono) and neutrophil (Neu) and eventual occurrence of pulmonary edema and mortality (Stoll et al., 2023). By combining single-cell RNA sequencing (scRNA-seq) and spatial transcriptomics assays, we depicted the dynamically changing inflammatory cell states in the DAD lung and identified an intricate interplay between hyper-inflammatory fibroblast (Fib) and Neu (Su et al., 2023). In addition, the deficiency of growth differentiation factor 15 (GDF15) was found to exacerbate the inflammation, indicating its regulatory role during DAD (Deng et al., 2022).

In this follow-up study, through an integrated exploitation of scRNA-seq, parabiosis, adoptive transfer, cytometry by time of flight (CyTOF), classical flow cytometry (FCM), multi-color immunohistochemistry (mIHC), and enzyme-linked immunosorbent assay (ELISA) techniques, we depicted the developmental trajectories of IM^pi^ and IM^ai^ from recruited Mono^pi^. Especially, a Mono^pi^ subset was shown to acquire the proliferating phenotype (namely pMono^pi^), which was driven by GDF15, and moreover pMono^pi^ served as a precursor for IM^ai^ generation. Collectively, this study would provide new insights into the underlying cellular mechanisms for Mono-to-IM transition in the DAD lung, offering a theoretical basis for developing novel therapeutic strategies against acute lung injury.

## Results

### Accumulation of Mono^pi^ and IM in the DAD lung

**Figure 1.**
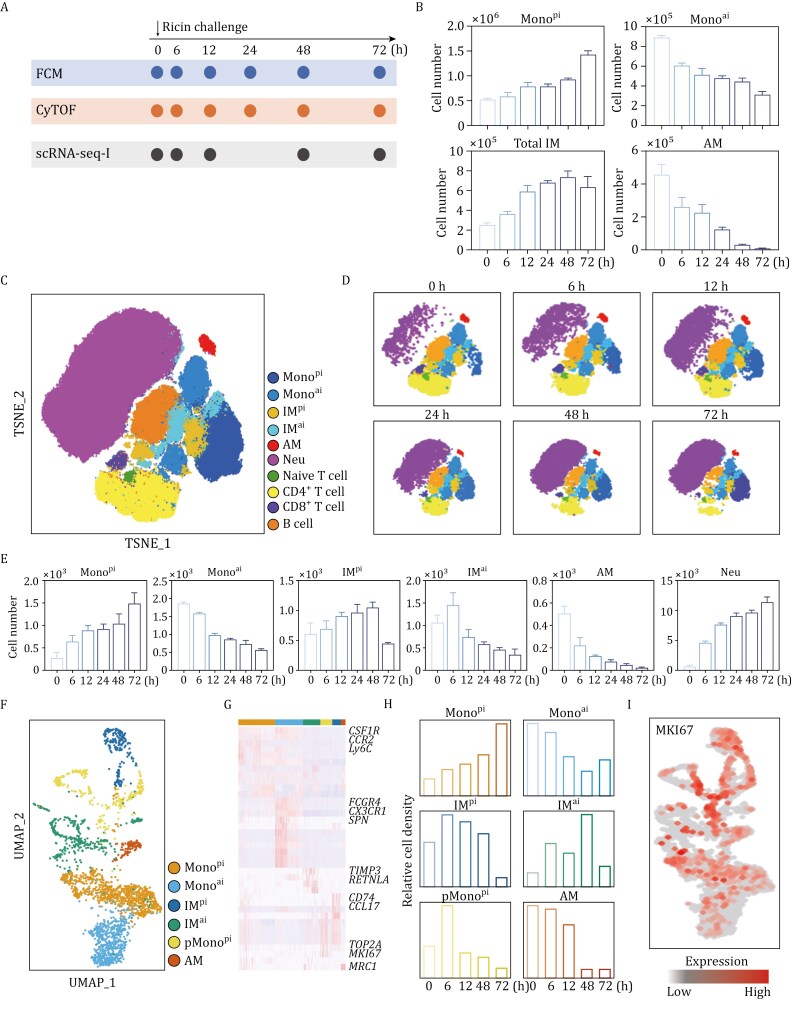
**Typing of immune cells in the DAD lung.** (A) Schematic illustration of experimental design. (B) FCM-based count of indicated cell types (*n* = 3). (C) CyTOF-based *t*-SNE visualization of major immune cell populations. (D) CyTOF-based *t*-SNE plots split by DAD timeline. (E) CyTOF-based count of various cell clusters (*n* = 3). (F) scRNA-seq-I-based UMAP plot of various cell clusters. (G) scRNA-seq-I-based heatmap of cell cluster-specific upregulated genes. Rows and columns denote genes and cell clusters, respectively. (H) scRNA-seq-I-based histograms of relative count of various cell clusters. (I) scRNA-seq-I-based expression pattern of MKI67 in various cell clusters. Data are presented as mean ± SD.

Based on our previous mouse model of ricin-induced DAD (Deng et al., 2022; Su et al., 2023), we performed multiple experiments on mononuclear phagocyte (MNP) samples sorted from the lung tissues at various hours post ricin challenge (Fig. 1A). First, FCM experiments (Figs. 1B and S1) revealed an increasing trend in the numbers of bulk IM (MerTK^+^CD64^+^CD11b^+^) and Mono^pi^, while demonstrating a decrease in Mono^ai^ and alveolar macrophage (AM, MerTK^+^ CD64^+^CD11c^+^CD11b^−^). This was consistent with the fact that AM, as a target cell, eliminated rapidly after lethal ricin inhalation (Su et al., 2023). Second, CyTOF experiments (Figs. 1C and S2) disclosed a high degree of heterogeneity in MNP population. Dimensionality reduction via *t*-distributed Stochastic Neighbor Embedding (*t*-SNE) analysis enabled the classification of major immune cell populations into 10 distinct clusters, particularly including Mono^pi^, Mono^ai^, IM^pi^, IM^ai^, AM, and Neu (CD45^+^CD11b^+^Ly6G^+^). As shown in Fig. 1D–E, both CyTOF and FCM experiments detected the generally consistent trends for Mono^pi^, Mono^ai^, and AM. While in CyTOF, the total IM was divided into IM^pi^ and IM^ai^ subsets, revealing a significant increase in IM^pi^ and a corresponding decrease in IM^ai^. These results indicated that IM response in the DAD lung was dominated by inflammatory phenotypes and primarily driven by IM^pi^ expansion. As expected, the rapid and massive influx of Neu to the lung was a hallmark of acute inflammation upon DAD, while the increasing trends for Mono^pi^ and IM^pi^ indicated a pro-inflammatory response in the DAD lung. Third, MNP samples were subjected to scRNA-seq-I experiments. Using defined functional markers as shown in Fig. S3A, we identified a total of 6 populations, namely Mono^pi^, Mono^ai^, IM^pi^, IM^ai^, pMono^pi^ (CD11b^+^Ly6C^hi^CCR2^hi^CX3CR1^lo^MKI67^+^TOP2A^+^), and AM (Fig. 1F and 1G). Generally, scRNA-seq-I experiments confirmed the increasing trends for Mono^pi^, IM^pi^, and IM^ai^, and the decreasing trends for Mono^ai^ and AM. Notably, the newly identified pMono^pi^ was highly enriched for expression of the cell proliferation marker MKI67 (Ma et al., 2022) and significantly accumulated in the DAD lung (Fig. 1G–I). Fourth, as revealed by gene enrichment analysis of scRNA-seq-I data, Mono^pi^ and Mono^ai^ shared overlapping immunoregulatory functions (e.g., “leucocyte migration”, “cytokine-mediated signaling pathway”, and “regulation of immune effector process”), whereas IM^pi^ and IM^ai^ were enriched with pro-inflammatory features (e.g., “response to IFN-γ”, and “regulation of cell killing”) and anti-inflammatory functions (e.g., “surfactant homeostasis”, and “lung development”), respectively (Fig. S3B). Collectively, this combined FCM, CyTOF, and scRNA-seq approach highlighted the accumulation of Mono^pi^, IM^pi^, and IM^ai^ in the DAD lung.

### Circulating Mono as an origin of accumulated IM in the DAD lung

**Figure 2.**
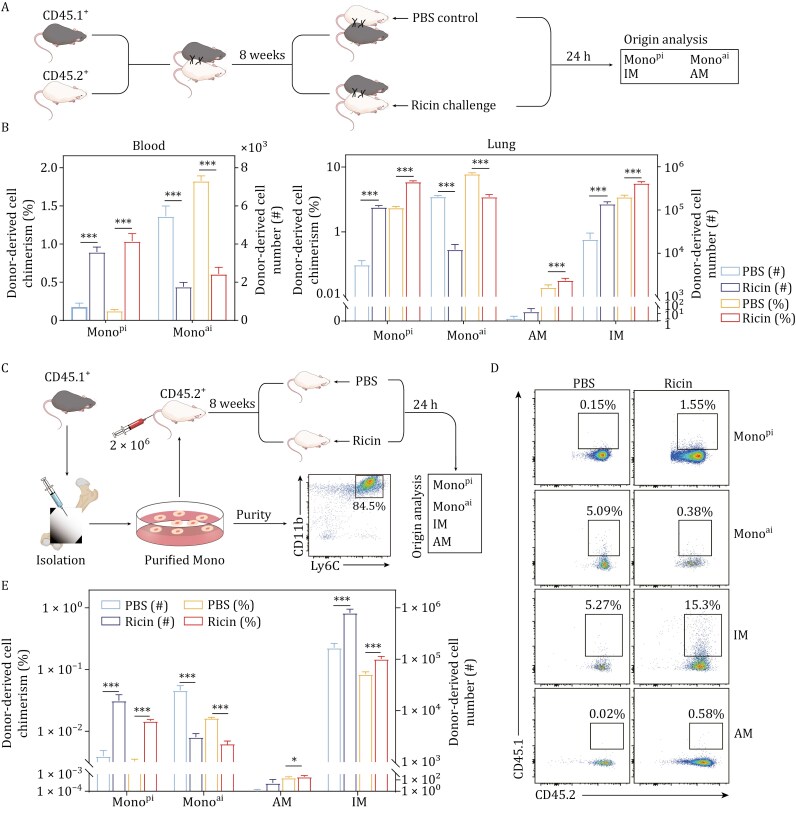
**Circulating Mono as an origin of accumulated IM in the DAD lung.** (A) Schematic workflow of parabiosis experiments. Mice are challenged with PBS or ricin. Contribution of CD45.1^+^ donor-derived cells to Mono^pi^, Mono^ai^, IM, and AM is analyzed. (B) Parabiosis-based chimerism and cell count of CD45.1^+^ donor-derived cells in CD45.2^+^ recipient mice. Mono^pi^ and Mono^ai^ are analyzed in the blood, while Mono^pi^, Mono^ai^, IM, and AM are analyzed in the lung. (C) Schematic workflow of adoptive transfer experiments. FCM validates the purity of isolated Mono from CD45.1^+^ mice. (D and E) Adoptive transfer-based chimerism and cell count of CD45.1^+^ donor-derived Mono^pi^, Mono^ai^, IM, and AM in the lung of CD45.2^+^ recipient mice, *n* = 4. Data are presented as mean ± SD. ns: *P *≥ 0.05, *P < 0.05, ***P < 0.001, Student’s *t*-test. The elements in (A) and (C) were created using Adobe illustrator 2022 software.

To determine the origin of accumulated IM in the DAD lung, we performed complementary parabiosis and adoptive transfer experiments using CD45.1^+^ (donor) and CD45.2^+^ (recipient) mice. First, in parabiosis experiments, we surgically joined CD45.1^+^ and CD45.2^+^ mice to establish shared circulation. After 8-week chimerism establishment, recipient (CD45.2^+^) mice were challenged with ricin, allowing assessment of donor-derived cell replenishment (Fig. 2A). In recipient mice, IM exhibited a significantly higher percentage and amount of chimerism in the DAD lung compared to non-injured control, highlighting DAD-induced Mono-to-IM transition in the lung; moreover, an analysis within Mono population revealed a significant increase in the amount of Mono^pi^ in the DAD lung, while Mono^ai^ exhibited an opposite trend, suggesting that Mono^pi^ was more predisposed to engrafting into the DAD lung (Fig. 2B). Similar trends for Mono^pi^ and Mono^ai^ were also evidenced in the blood (Fig. 2B), indicating that Mono^pi^ rather than Mono^ai^ preferably moved to the recipient mouse during DAD. By contrast, donor-derived AM showed minimal exchange between the peripheral circulation and the local lung, with a significantly lower count of chimerism relative to IM, suggesting that AM was primarily self-renewed (Fig. 2B). These findings implied that IM, but not AM, was primarily replenished from circulating Mono. Second, in adoptive transfer experiments, we sorted CD45.1^+^ Mono samples and intravenously transferred them into CD45.2^+^ recipients before challenge. The results revealed a significantly higher proportion of donor-derived IM in the lung compared to the blank control, further supporting circulating Mono as the origin of accumulated IM during DAD (Fig. 2C). The changing trends for Mono^pi^, Mono^ai^, and AM detected by adoptive transfer experiments here (Fig. 2D and 2E) were generally consistent with those determined by parabiosis experiments above. Additionally, we observed that CD11b^+^ dendritic cell (DC) exhibited a higher percentage of chimerism compared to CD11b^−^ DC (Fig. S4), confirming the monocytic ontogeny of CD11b^+^ DC during DAD. Collectively, this combined parabiosis and adoptive transfer strategy confirmed that circulating Mono serve as the origin of accumulated IM in the DAD lung, although the specific subsets of Mono and IM could not be fully defined here.

### Emergence of pMono^pi^ in the DAD lung

**Figure 3.**
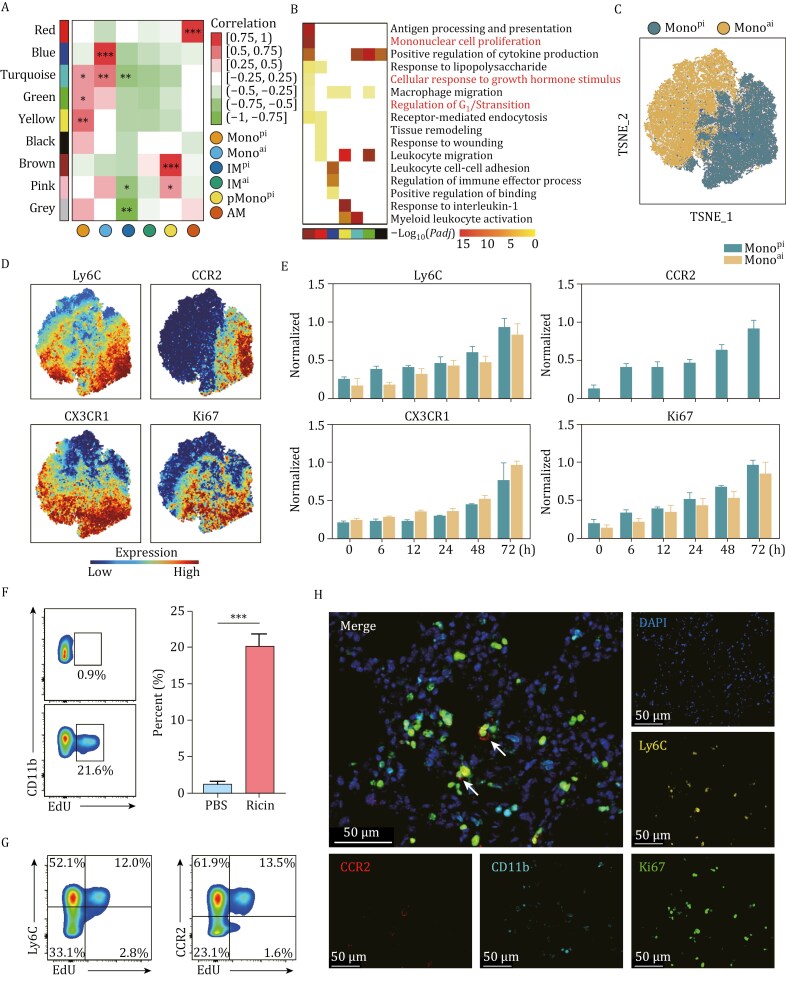
**Emergence of pMono** ^**pi**^  **in the DAD lung.** (A) scRNA-seq-I-based heatmap of module-cluster weight correlations and corresponding *P* values from WGCNA. Rows and columns correspond to co-expression modules and cell subsets, respectively. Color scales (right) show module-cluster correlations from −1 (green) to 1 (red). (B) scRNA-seq-I-based heatmap of enriched biological processes of genes in each module from WGCNA. (C) CyTOF-based dotplot of Mono^pi^ and Mono^ai^. (D) CyTOF-based dotplots of the expression level of cell markers Ly6C, CCR2, CX3CR1, and Ki67 in CyTOF. (E) CyTOF-based histograms of normalized expression levels of Ly6C, CCR2, CX3CR1, and Ki67 in Mono^pi^ and Mono^ai^ (*n* = 3). (F) Representative FCM images (left) detecting pMono^pi^ (CD11b^+^Ly6C^+^ CCR2^+^EdU^+^), and corresponding statistical analysis (right). Mice are challenged with PBS or ricin. Pre-gated on live CD45^+^CD11b^+^CD3^−^ NK1.1^−^CD19^−^Ly6G^−^, *n* = 3. Data are presented as mean ± SD. *P < 0.05, **P < 0.01, ***P < 0.001, Student’s *t*-test. (G) Representative FCM images showing expression profiles of Ly6C and CCR2 in pMono^pi^. (H) Representative mIHC images with staining of CD11b, Ly6C, CCR2, and Ki67 to detect pMono^pi^ (white arrows).

A sequence of data mining and experimental validation procedures was conducted to elucidate whether recruited Mono^pi^ could reenter the mitotic cycle in the DAD lung. First, Weighted gene co-expression network analysis (WGCNA) was performed on scRNA-seq-I data to identify genes with correlated expression patterns, followed by analysis of the correlation between the identified modules and annotated cell clusters for selection of modules significantly associated with specific cell types. This analysis identified a total of 9 distinct co-expression modules (Fig. 3A), which exhibited distinct and pronounced up-regulation states and corresponded to the above-defined cell subsets. Second, gene ontology (GO) enrichment analysis of the above modules revealed distinct biological functions (Fig. 3B). The yellow module was highly related to Mono^pi^ with enriched pro-inflammatory terms such as “leukocyte migration” and “response to IL-1.” The blue module was highly associated with Mono^ai^, with enriched anti-inflammatory terms such as “cell-cell adhesion” and “regulation of immune effector processes”. The brown module was highly related to pMono^pi^ with enriched regulatory terms such as “antigen processing and presentation”, “positive regulation of cytokine production,” and “macrophage migration.” Notably, this brown module also exhibited the enrichment of cell proliferation terms such as “mononuclear cell proliferation” and “regulation of G_1_/S transition of mitotic cell cycle.” Third, CyTOF data were used to separately analyze Mono^ai^ and Mono^pi^ (Fig. 3C). Mono^ai^ highly expressed CX3CR1, while Mono^pi^ highly expressed Ly6C and CCR2, resembling features of IM^pi^. The expression level of Ki67 in Mono^pi^ was significantly higher than that in Mono^ai^ (Fig. 3D and 3E), suggesting a proliferating phenotype within Mono^pi^. Fourth, by using the 2 classic cell proliferation markers Ki67 (Ma et al., 2022) and EdU (Khan and Robbins, 2023), FCM (Fig. 3F and 3G) and mIHC (Fig. 3H) experiments confirmed the presence of pMono^pi^ (CD11b^+^Lin^−^Ly6C^+^CCR2^+^EdU^+^ for FCM, and CD11b^+^Ly6C^+^CCR2^+^Ki67^+^ for mIHC) in the DAD lung. Collectively, these findings showed that pMono^pi^, as a subpopulation of Mono^pi^, had evolved to acquire a proliferating phenotype in the DAD lung.

### pMono^pi^ as the precursor of accumulated IM^ai^ in the DAD lung

**Figure 4.**
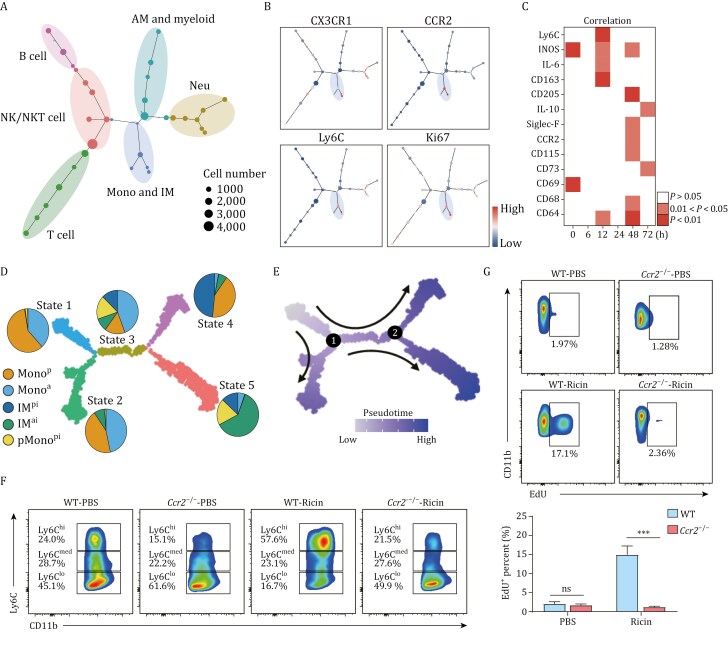
**pMono** ^**pi**^  **contributes to IM**^**ai**^  **development in the DAD lung.** (A) SPADE analysis of CyTOF. Cell subpopulations are annotated manually in circles. Node sizes represent cell counts. (B) Expression levels of CX3CR1, CCR2, Ly6C, and Ki67 in (A). (C) CyTOF-based heatmap showing correlation of Ki67 expression with individual lineage markers. (D) scRNA-seq-I-based monocle analysis depicting the 5 developmental states. Proportion pies show cell distribution of each cluster across each state. (E) scRNA-seq-I-based pseudotime assignment of monocle trajectory analysis. (F) Representative FCM images detecting Ly6C expression in WT and *Ccr2*^−/−^ mice at 48 h. Mice are challenged with PBS or ricin. Pre-gated on live CD45^+^CD11b^+^CD3^−^NK1.1^−^CD19^−^Ly6G^−^. Numbers indicate cell frequencies of Ly6C^hi^ (Mono^pi^), Ly6C^med^, and Ly6C^lo^ Mono subsets. (G) Representative FCM images (upper) detecting pMono^pi^ (CD11b^+^Ly6C^+^CCR2^+^EdU^+^) in WT and *Ccr2*^−/−^ mice at 48 h, and corresponding statistical analysis (down). Mice are challenged with PBS or ricin. Pre-gated on live CD45^+^CD11b^+^CD3^−^NK1.1^−^CD19^−^Ly6G^−^. *n* = 4. Data are presented as mean ± SD. ns: *P* ≥ 0.05, ***P < 0.001, Student’s *t*-test.

The relationship between pMono^pi^ and IM^ai^ in the DAD lung was systematically characterized. First, to characterize the relationships between individual cell populations and construct cellular differentiation trajectories in lung immune cells, we performed spanning-tree progression analysis of density-normalized events (SPADE) analysis on CyTOF data based on the lineage-specific markers (Fig. 4A). SPADE analysis revealed an overlapping expression of key markers Ki67, CX3CR1, Ly6C, and CCR2 between Mono and IM (Fig. 4B). Further correlation analysis showed a high degree of co-expression of these markers (Fig. 4C), indicating a topological connection between Mono and IM. Second, to investigate the transition of Mono and IM at single-cell resolution, we employed Monocle pipeline for pseudo-temporal trajectory inference and assessed differentiating potential with CytoTRACE. With Monocle, the overall clusters comprising Mono and IM were assigned into 5 states (Fig. 4D). State 1 was highly associated with Mono^pi^ and had the highest differentiating potential, suggesting that Mono^pi^ would represent the starting point of Mono-to-IM transition (Figs. 4D and S5). By contrast, State 5 was related to IM^ai^ and had the lowest differentiating potential, indicating that IM^ai^ would represent the terminal point of this transition (Figs. 4D and S5). Gene expression of specific differentiation marker genes yielded similar results (Fig. S6). Third, pseudotime trajectory analysis was performed on scRNA-seq-I data to organize cells into a progression of sequential maturation stages (Fig. 4E). Gene expression analysis along pseudotime revealed significant and robust changes at branch points 1 and 2 (Figs. 4E, S7A and S7B). Enrichment analysis showed that terms related to “mononuclear cell proliferation” were significantly enriched around these branch points (Fig. S7B, red box), highlighting the critical role of pMono^pi^ in determining cell fate and commitment during Mono-to-IM transition. Fourth, CCR2-deficient (*Ccr2*^−/−^) mice were utilized to identify the specific origin of pMono^pi^. As expected, *Ccr2*^−/−^ mice exhibited a significantly reduced number of Ly6C^hi^ Mono^pi^ in the lung compared to Wild-type (WT) littermates (Fig. 4F), suggesting that CCR2 deficiency specifically impaired the recruitment of Mono^pi^. The absence of CCR2 was also associated with a marked reduction of pMono^pi^ in the DAD lung (Fig. 4G). These findings strongly supported the notion that recruited Mono^pi^ served as the precursor of pMono^pi^ in the DAD lung. Collectively, upon entering the lung tissue from the blood, mature non-proliferating Mono^pi^ cells would be partially converted into a proliferating state that served as the precursor of accumulated IM^ai^ in the DAD lung, achieving the developmental trajectory Mono^pi^-to-pMono^pi^-to-IM^ai^.

### Involvement of GDF15 in Mono-to-IM transition in the DAD lung

**Figure 5.**
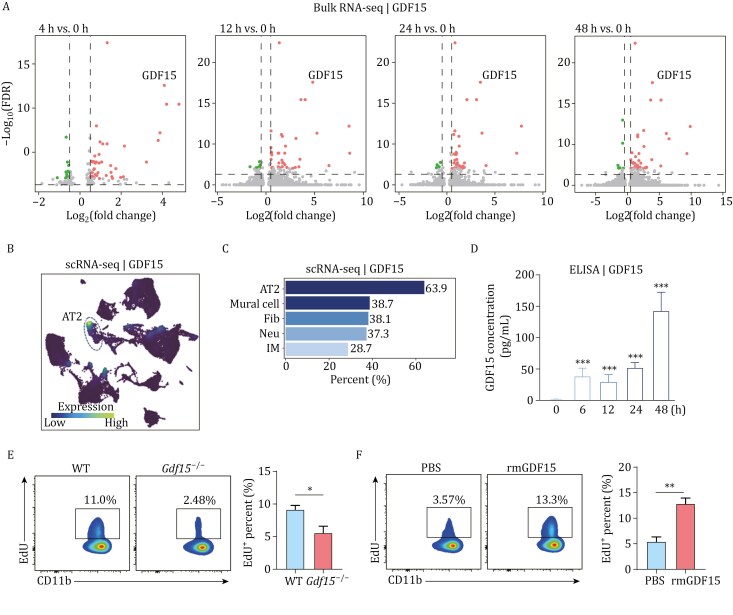
GDF15 as a modulator driving pMono^pi^ differentiation in the DAD lung. (A) Bulk RNA-seq-based volcano plots of DEGs. (B) scRNA-seq-based UMAP plots of cell type expressing GDF15 transcripts. (C) scRNA-seq-based bar plot of the top 5 cell types expressing GDF15. (D) ELISA-based histograms of GDF15 concentrations in BALF (*n* = 3). ***P < 0.001, one-way ANOVA with Tukey’s multiple comparisons test. (E) Representative FCM images (left) detecting pMono^pi^ (CD11b^+^Ly6C^+^CCR2^+^EdU^+^) in ricin-challenged WT and *Gdf15*^−/−^ mice, and corresponding statistical analysis (right). (F) Representative FCM images (left) detecting pMono^pi^ (CD11b^+^Ly6C^+^CCR2^+^EdU^+^) in ricin-challenged *Gdf15*^−/−^ mice followed by intratracheal administration with PBS or rmGDF15, and corresponding statistical analysis (right). (E and F) Pre-gated on live CD45^+^CD11b^+^CD3^−^NK1.1^−^CD19^−^Ly6G^−^. *n* = 3. Data are presented as mean ± SD. *P < 0.05, **P < 0.01, Student’s *t*-test.

We then moved to identify the key modulator driving the development of pMono^pi^ during DAD. First, we re-analyzed our previous RNA-seq (Jiao et al., 2021) and scRNA-seq data (Su et al., 2023). RNA-seq showed an up-regulation of *Gdf15* gene expression (Fig. 5A), while scRNA-seq indicated alveolar type 2 cells (AT2) as the predominant source of GDF15 expression during DAD (Fig. 5B and 5C). Second, we confirmed the increased level of GDF15 protein in bronchoalveolar lavage fluid (BALF) samples using ELISA (Fig. 5D), confirming the inducible production of GDF15 in the lung tissues upon DAD. Third, FCM experiments demonstrated a reduced percentage of pMono^pi^ in the DAD lung of GDF15-deficient (*Gdf15*^−/−^) mice compared to WT (Fig. 5E). Fourth, after aerosolized intratracheal administration of recombinant GDF15 (rmGDF15) protein in ricin-challenged *Gdf15*^−/−^ mice, an increase in the percentage of pMono^pi^ was observed relative to PBS administration (Fig. 5F). Taken together, GDF15 would act as a modulator driving pMono^pi^ differentiation in the DAD lung.

**Figure 6.**
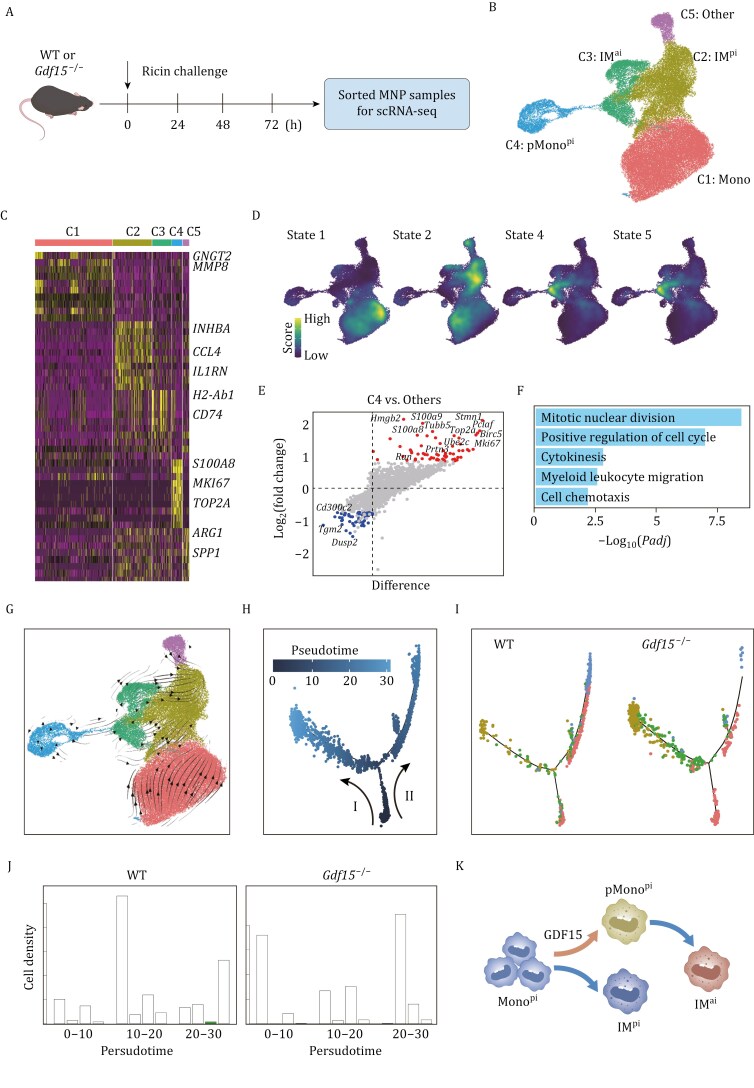
**GDF15 as a modulator driving Mono-to-IM transition in the DAD lung.** (A) Schematic illustration of scRNA-seq-II experimental design. (B) UMAP plot of various cell clusters. (C) scRNA-seq-II-based heatmap of cell cluster-specific upregulated genes. Rows and columns denote genes and cell clusters, respectively. (D) scRNA-seq-II-based signature score of monocle states in Fig. 4D. (E) scRNA-seq-II-based volcano plots of up- (red) and down-regulated (blue) genes in C4. (F) scRNA-seq-II-based functional enrichment of up-regulated genes in C4. (G) RNA velocity analysis showing transition potential among cell clusters. Arrows predicate directions of transition. (H) Pseudotime analysis demonstrating 2 distinct developmental trajectories starting from Mono. (I) Mapping of cell clusters in (B) from WT or *Gdf15*^−/−^ mice onto inferred trajectory in (G). (J) Histogram showing cell densities calculated by aggregating cells into bins (bins = 30) along pseudotime. (K) Schematic model of Mono-to-IM developmental trajectories. The elements in (A) and (K) were created using Adobe illustrator 2022 software.

To explore the defined biological roles of GDF15, we performed scRNA-seq-II experiment (Fig. 6A) with MNP samples sorted from the DAD lung tissues of *Gdf15*^−/−^ and WT mice (see Fig. S8 for sorting strategy). The total of 21,819 sequenced cells could be categorized into 5 distinct clusters (Fig. 6B and 6C), and these 5 clusters were then attributed to the 5 developmental states from scRNA-seq-I (Fig. 4D), revealing the high data coherence between these scRNA-seq-II and scRNA-seq-I (Fig. 6D). The subsequent data mining of scRNA-seq-II was centered on the role of GDF15 in Mono-to-IM transition. First, gene expression analysis revealed that pMono^pi^ were enriched for not only the proliferating markers MKI67 and TOP2A (Uuskula-Reimand et al., 2022) but also the biological processes related to mitotic division and leukocyte migration (Fig. 6E and 6F). Second, there was a significant reduction in the proportion of pMono^pi^ among the total sequenced cells in the DAD lung of *Gdf15*^−/−^ mice compared to WT, particularly at 48 h (Fig. S9A and S9B). Third, applying RNA velocity, a method inferring precursor progeny cell dynamics, we observed a clear differentiation directionality from pMono^pi^ to IM^ai^ (Fig. 6G), indicating that pMono^pi^ served as the precursor cell giving rise to Mono-derived IM^ai^. Fourth, as shown by pseudotime trajectory analysis, there were 2 major developmental trajectories: Mono-to-IM^pi^ in trajectory I, and Mono-to-pMono^pi^ in trajectory II (Fig. 6H and 6I); moreover, pMono^pi^ would enhance the side-wind trajectory of IM^ai^ repopulation in WT mice compared to *Gdf15*^−/−^ mice (Fig. 6I and 6J). In summary, GDF15 facilitated Mono^pi^-to-pMono^pi^-to-IM^ai^ transition in the DAD lung (Fig. 6K).

## Discussion

**Figure 7.**
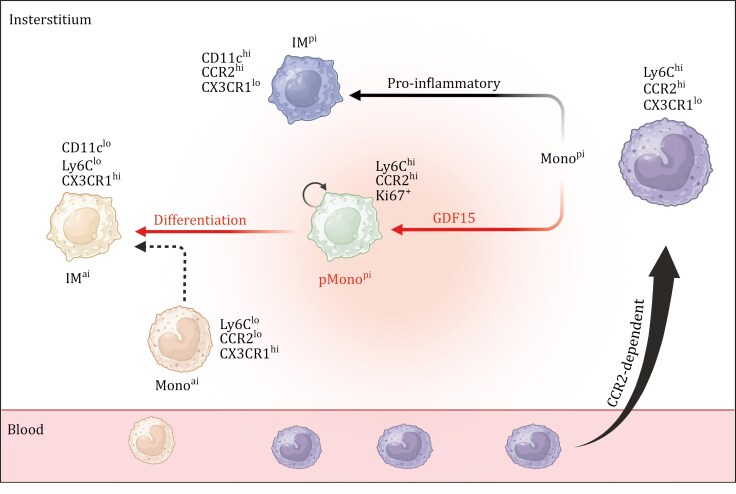
**Schematic illustration of Mono-to-IM transition during ricin-induced DAD.** Lethal ricin inhalation will induce the development of 2 distinct IM subsets, namely IM^pi^ and IM^ai^, from recruited Mono^pi^ in the DAD lung. Notably, a subset of recruited Mono^pi^, designated pMono^pi^, can get the proliferating phenotype, and meanwhile serve as the intermediate of Mono^pi^-to-IM^ai^ transition. GDF15 facilitates Mono^pi^-to-pMono^pi^-to-IM^ai^ transition. The figure was created with BioRender.com.

Cellular and molecular mechanisms involved in DAD pathogenesis remain largely unclear. By employing a mouse model of DAD induced by lethal ricin inhalation, the current study analyzed the intricate processes that drive the differentiation of recruited Mono into IM subsets in the DAD lung (Fig. 7). Mono^pi^ was intensively recruited from the blood into the lung in a classic CCR2-dependent manner and would further transit into IM^pi^ and IM^ai^, with a significant increase of IM^pi^ alongside a moderate rise of IM^ai^. Furthermore, our study identified a proliferating Mono^pi^ subset (pMono^pi^) that functioned as the intermediate of Mono^pi^-to-IM^ai^ transition (Fig. 7). Local signals significantly influenced Mono behaviors in inflammatory tissues (Mass et al., 2023). Recruited Mono would play a crucial role in maintaining the balance of IM^pi^ and IM^ai^ in the DAD lung, and it could differentiate into Mono^ai^, Mono^pi^, and pMono^pi^. The high plasticity nature of Mono was in alignment with tissue-specific needs (Adams et al., 2025; Guilliams et al., 2018, 2021). Compared to its typical Mono^pi^ counterpart, pMono^pi^ was at an intermediate transiting state and exhibited the relatively reduced expression of classical pro-inflammatory Mono^pi^ marker Ly6C and the augmented expression of anti-inflammatory IM^ai^ marker CX3CR1.

Mono^pi^ was a major circulating Mono subpopulation derived from bone marrow (Trzebanski et al., 2024), and had been identified as a critical precursor for tissue macrophage replenishment (Moore et al., 2023; Teh et al., 2022; Vanneste et al., 2023). Among lung MNP populations, AM primarily underwent local proliferation for self-renewal, whereas the IM pool relied predominantly on recruited Mono^pi^ (Hou et al., 2021). Notably, our study further revealed that IM exhibited a significantly higher cellular turnover rate than AM after lung injury, where IM underwent substantial expansion via Mono^pi^ differentiation. Although Mono^pi^ remained non-proliferative in circulation (Ong et al., 2018; Pang et al., 2023), it would acquire proliferating capacity upon migration into the lung tissues, which was a prerequisite for its differentiation into IM^ai^. As demonstrated by these findings aligned with the emerging evidence (Pang et al., 2020; Rolot et al., 2019; Vanneste et al., 2023), specific Mono^pi^ subsets (e.g., pMono^pi^) could undergo a cascade differentiation process, including a proliferating phase, and ultimately form functionally specialized macrophage effector populations (e.g., IM^ai^).

GDF15, a member of TGF-β superfamily, was an immune regulator with many functions, and GDF15 signaling offered a defense against the excessive inflammation induced by tissue injuries (Assadi et al., 2020). As demonstrated in this study, the presence of GDF15 facilitated Mono^pi^-to-pMono^pi^-to-IM^ai^ transition, whereas a deficiency in GDF15 impaired the developmental trajectory towards IM^ai^ and meanwhile promoted a negative feedback shift to IM^pi^, suggesting a pivotal role of GDF15 in navigating Mono^pi^-to-pMono^pi^-to-IM^ai^ transition. Previous studies revealed that GDF15 exerted anti-inflammatory effects and restored macrophage to M2-like polarization in injury diseases (Deng et al., 2022; Li et al., 2018; Luan et al., 2019; Pereiro et al., 2020). During myocardial infarction, GDF15 could inactivate inflammatory pathways in regulatory T cells and increase macrophage M2 polarization to improve cardiac function (Libby, 2021; Takaoka et al., 2024). Similarly, in tumor microenvironments, GDF15 overexpression induced the M2-like polarization of macrophage, while inhibition of CCR2-dependent Mono^pi^ recruitment reduced tumor metastasis (Xu et al., 2021). To the best of our knowledge, this is the first report regarding the driving modulatory action of GDF15 on Mono-to-IM transition in the context of DAD. GDF15 would represent a candidate target for treating acute lung injury diseases.

To date, there are no clinically approved post-exposure medical countermeasures against ricin intoxication, but some immunomodulatory drugs have shown promise in reducing lung injury and improving patient outcomes (Gal et al., 2017; Rasetti-Escargueil et al., 2023). Therefore, it is essential to clarify the cellular and molecular mechanisms involved in ricin inhalation to develop new and targeted therapeutic strategies aimed at reducing morbidity and mortality. This study presents the cellular and molecular immunological atlas in the DAD lung after ricin inhalation, which is the most lethal route of ricin intoxication. The findings from this research could significantly inform public health initiatives and improve environmental safety protocols. Future research into molecular mechanisms by which GDF15 influences Mono/IM differentiation holds great promise for developing novel therapeutic strategies for treating acute lung injury as well as other inflammatory diseases.

## Supplementary Material

pwaf070_Supplementary_Materials_1

## Data Availability

The accession number for scRNA-seq is GEO: GSE161524. The DOI for bulk RNA-seq is 10.1016/j.toxlet.2020.11.012.

## Code availability

The bioinformatics analyses were conducted according to established tutorials, and no specific custom script or code was used for data analysis.

## Artificial intelligence (AI)

No AI tools or technologies were used in the research process or manuscript preparation.
