# Supplementary material for: A cell differentiation landscape for monocyte and interstitial macrophage in the lung with diffuse alveolar damage
Source: Protein Cell. 2025 Aug 4;17(1):46–58. doi: 10.1093/procel/pwaf070 (PMC12888921; doi:10.1093/procel/pwaf070)
Supplement: pwaf070_Supplementary_Materials_1 [file pwaf070_supplementary_materials_1.pdf]

Supporting Information

A cell differentiation landscape for monocyte and interstitial macrophage in the lung with diffuse alveolar damage

Duo Su[a,b,#], Mengyun Deng[a,#], Lingfei Hu[a], Hao Xie[a], Bo Yang[b], Huiying Yang[a,*], Dongsheng Zhou[a,*]

[a] State Key Laboratory of Pathogen and Biosecurity, Academy of Military Medical Sciences, Beijing 100071, China
[b] Reproductive Genetics Center, Bethune International Peace Hospital, Shijiazhuang 050082, China

[#] These authors contributed equally to this work.
* Corresponding authors: yhy324@aliyun.com (H. Yang); zhouds@bmi.ac.cn, dongshengzhou1977@gmail.com (D. Zhou).

Contents

Materials and methods 3
Fig. S1 .... 错误!未定义书签。
Fig. S2 .... 错误!未定义书签。
Fig. S3 .... 错误!未定义书签。
Fig. S4 .... 错误!未定义书签。
Fig. S5 .... 错误!未定义书签。
Fig. S6 .... 错误!未定义书签。
Fig. S7 .... 错误!未定义书签。
Fig. S8 .... 错误!未定义书签。
Fig. S9 .... 错误!未定义书签。
Table S1.. 错误!未定义书签。
Table S2.. 错误!未定义书签。
Table S3.. 错误!未定义书签。
Table S4.. 错误!未定义书签。
Table S5.. 错误!未定义书签。
Table S6.. 错误!未定义书签。
Table S7.. 错误!未定义书签。
References 20

Materials and methods

Mice

Female mice aged 6 to 10 weeks were used in all experiments. WT C57BL/6J mice were purchased from HFK Bio-Technology (China). Congenic $CD45.1^+$ and $CD45.2^+$ mice, along with *Ccr2*$^{-/-}$mice, were purchased from SMOC (China). *Gdf15*$^{-/-}$ mice were purchased from GemPharmatech (China). All genetically modified mouse strains were in C57BL/6J background.

Model of ricin-induced DAD

Ricin-induced DAD model was established in mice via aerosolized intratracheal inoculation as previously described (Su et al., 2023). Briefly, HRH-HAG5 MicroSprayer (Huironghe, China) was used to deliver a $1.5\times LD_{50}$ dose of ricin (~7.5 μg/kg) in 50 μL PBS into the lung of each mouse. Control mice received an equal volume of PBS. For rmGDF15 rescue assay, *Gdf15*$^{-/-}$ mice were inoculated with ricin as above and then randomly divided into the following 2 groups: one group received aerosolized intratracheal administration of rmGDF15 (400 ng/mouse, CAT# HY-P77945, MedChemExpress, USA) at 1 h post ricin challenge, while another group received PBS as a control.

FCM assay

To profile the dynamics of MNP populations, the lung of WT mice were collected at 0, 6, 12, 24, 48, and 72 h post ricin challenge. Single-cell suspensions were prepared by enzymatic digestion using 1.5 mg/mL collagenase A (CAT# 10103586001, Sigma-Aldrich, USA), 0.4 mg/mL DNase I (CAT# D5025, Sigma-Aldrich, USA), and 1.5 U/mL dispase II (CAT# D4693, Sigma-Aldrich, USA) in 10 mM HEPES buffer with 10% FBS (CAT# 10099141C, Thermo Fisher Scientific, USA) at 37 °C for 30 min, followed by sequential 70 μm filtration and centrifugation at 400×g for 5 min at 4 °C. Erythrocyte lysis was performed using ACK lysing buffer (CAT# A10492-01, Thermo Fisher Scientific, USA) for 2 min at room temperature and washed twice with ice-cold PBS. Viable cells were resuspended in PBS at 2×10^6 cells per 100 μL for sequential incubation: Fc-block (anti-CD16/32, CAT# 553141, BD Biosciences, USA) for 10 min at 4 °C, Horizon Fixable Viability Stain 510 (CAT# 564406, BD Biosciences, USA), and fluorochrome-conjugated antibodies, as listed in Table S1, for 30 min at 4 °C. Samples were analyzed on FACSymphony A5 flow cytometer (BD Biosciences, USA).

To confirm the presence of $pMono^{pi}$, each mouse received intraperitoneal injection of 5-ethynyl-2'-deoxyuridine (EdU, 50 mg/kg, CAT# A10044, Thermo Fisher Scientific, USA) at 36 h post ricin challenge, and the lung were collected at 48 h. Single-cell suspensions from the lung were surface-stained (Table S2), followed by intracellular EdU detection using Click-iT Plus EdU Alexa Fluor 488 Kit (CAT# C10337, Thermo Fisher Scientific, USA). Samples were analyzed on LSRFortessa flow cytometer (BD Biosciences, USA). Data were analyzed using FlowJo software (v10.8.1).

CyTOF assay

To disclose the heterogeneity in MNP populations, the lung of WT mice were collected at 0, 6, 12, 24, 48, and 72 h post ricin challenge. Single cell suspensions were prepared as described above. Cells were labeled with Cell-ID Cisplatin-198Pt (CAT# 201198, Fluidigm, USA) for

viability assessment and barcoded using Cell-ID 20-Plex Pd Barcoding Kit (CAT# 201060, Fluidigm, USA). Surface antigen staining was conducted with antibody cocktails, as shown in Table S3, at room temperature for 30 min, followed by intracellular staining after methanol permeabilization at 4 °C for 15 min.

Data were acquired using Helios CyTOF2 instrument (Fluidigm, USA) and stored as FCS 3.0 files, and then initial quality control and preprocessing were performed on Cytobank platform (v7.3.0), including removal of non-immune cells, doublets, and cellular aggregates. Processed data were then imported into Cytofkit2 (v2.0.1) R package for downstream analyses.

Unsupervised clustering of immune cell populations was performed using FlowSOM algorithm (v 1.18.0) (Van Gassen et al., 2015) with meta-clusters generated based on marker expression profiles. Cell populations were visualized in two-dimensional space through *t*-SNE and annotated according to lineage-specific markers.

Cellular differentiation trajectories were modeled using SPADE. This analysis was performed with SPADE (v1.10.4) R package and CytoTree (v1.0.3) R package, implementing density-dependent down-sampling, hierarchical clustering, and minimum spanning tree construction. Final visualizations were generated using ggplot2 (v3.5.1) R package (Qiu et al., 2011; Dai et al., 2021).

scRNA-seq experiments

To characterize Mono and IM differentiation, the following 2 different scRNA-seq experiments were performed on MNP cells sorted from the lung: scRNA-seq-I for WT mice at 0, 6, 12, 48, and 72 h post ricin challenge, and scRNA-seq-II for WT and *Gdf15*$^{-/-}$ mice at 0, 24, 48, and 72 h post ricin challenge. Antibody information for scRNA-seq-I and scRNA-seq-II was provided in Table S4 and S5, respectively. All surface markers were stained for 30 min at 4 °C. Viable cells were identified using Horizon Fixable Viability Stain 510 and sorted on FACSAria SORP flow cytometer (BD Biosciences, USA). Sorted cells were resuspended at 1,000 cells per mL and loaded onto Next GEM chip (CAT# 1000127, 10×Genomics, USA). scRNA-seq libraries were prepared by 10×Genomics and Chromium Single Cell 30 Reagent Kit (CAT# 120237, 10×Genomics, USA), and unique cell hashtags antibodies were also used to label cells from each library individually. cDNA quality was assessed by G2939BA Bioanalyzer 2100 (Agilent, UK), and libraries were quantified by Qubit dsDNA HS kit (CAT# Q32851, Thermo Fisher Scientific, USA). Gene expression libraries were sequenced on NovaSeq 6000 System (Illumina, USA) with up to 100 GB of data per library.

scRNA-seq data mining

Raw data were processed using Cell Ranger (v3.0.2). Reads were demultiplexed, aligned to the GRCm38 mouse reference genome, and quantified into UMI counts. Quality control was performed using Seurat (v4.2.0) (Butler et al., 2018). Cells with fewer than 200 or more than 5,000 detected genes were removed. Cells with mitochondrial gene content exceeding 20% were excluded. Raw counts were log-normalized. Highly variable genes were identified. Batch effects were corrected using FindIntegrationAnchors and IntegrateData functions. Data were scaled and regressed against UMI counts and mitochondrial RNA percentages.

For cell clustering and annotation, principal component analysis was performed. Unsupervised clustering was conducted using the Louvain algorithm in Seurat (v4.2.0).

Clusters were visualized with UMAP. Cell types were annotated based on the top differentially expressed genes and known marker genes. Non-target cell clusters (e.g. Neu, T cell, and B cell) were removed. Only MNP data were retained for downstream analysis.

For GO enrichment analysis, DEGs were identified. Functional enrichment analysis was performed using clusterProfiler (v4.0). GO terms were examined. Significant terms (adjusted $P<0.05$) were selected. Biological processes, molecular functions, and cellular components were analyzed (Wu et al., 2021). In addition, Pseudotime analysis was performed using Monocle (v2.18.0) (Qiu et al., 2017a; Qiu et al., 2017b). Differentiation trajectories were constructed. A total of 5 cellular states (State 1 to 5) were defined. Top 50 DEGs per state (adjusted $P<0.05$) were identified. Cell states from scRNA-seq-I were mapped to scRNA-seq-II using AddModuleScore function in Seurat (v4.2.0).

RNA velocity was estimated using scVelo (v0.2.3). Spliced and unspliced mRNA ratios were calculated. Directed cell transitions were inferred. Velocity streams were visualized on UMAP plots. Results were compared with pseudotime trajectories (Bergen et al., 2020).

WGCNA was applied using the WGCNA R package (v1.69). Co-expressed gene modules were identified. Modules were correlated with cell clusters or differentiation states. Key functional modules were selected. Biological pathways were analyzed (Langfelder et al., 2008).

Parabiosis establishment and chimerism analysis

To investigate circulating cell recruitment, parabiosis experiments were performed using CD45.1$^+$ (donor) and CD45.2$^+$ (recipient) mice as previously described (Kamran et al., 2013). After 5-day ibuprofen pretreatment (30 mg/kg in drinking water), mice were anesthetized with 2% isoflurane and surgically joined through bilateral skin incisions from olecranon to knee joint, with joints fixed by 5-0 Prolene sutures (CAT# W9127, Ethicon, USA) and dermal layers closed by 4-0 Vicryl sutures (CAT# J304H, Ethicon, USA). Postoperative antibiotics (1 g/L ampicillin, 1 g/L neomycin, 1 g/L metronidazole, or 0.5 g/L vancomycin) were administered in drinking water for 4 weeks. At 8 weeks post-parabiosis, parabionts were randomly divided into the following 2 groups: CD45.2$^+$ mice receiving inoculation with ricin or PBS. 24 h later, the lung and blood samples were collected for FCM analysis of leukocyte chimerism. Chimerism was calculated as %CD45.1$^+$ / (%CD45.1$^+$ + %CD45.2$^+$) in CD45.2$^+$ mice. The antibody information was listed in Table S6.

Adoptive transfer assay

To investigate the recruitment of circulating Mono, adoptive transfer experiments were also performed using CD45.1$^+$ and CD45.2$^+$ mice. Bone marrow-derived CD45.1$^+$ Mono were isolated by negative selection using EasySep Mouse Monocyte Isolation Kit (CAT# 19861, StemCell Technologies, USA), with >80% purity (CD11b$^+$ Ly6C$^+$). CD45.1$^+$ Mono (2×10^6 cells in 200 μL PBS) were administered to CD45.2$^+$ recipients via tail vein injection. 8 weeks later, CD45.2$^+$ mice were subjected to intratracheal inoculation with ricin or PBS (as a control). 24 h later, the lung were collected for FCM analysis of leukocyte chimerism. Chimerism was calculated as above in parabiosis. The antibody information was listed in Table S6.

mIHC assay

To confirm the presence of $pMono^{pi}$, mIHC was performed using Opal 7-Color Manual IHC Kit (CAT# NEL811001KT, PerkinElmer, USA). Formalin-fixed paraffin-embedded lung sections (5 μm) were mounted on charged slides and baked at 60 °C for 1 h. Sections were deparaffinized in xylene (2×10 min) and rehydrated through a graded ethanol series (95%, 85%, and 75%, 5 min each). Antigen retrieval was performed in citrate buffer (pH 6.0) at 95 °C for 20 min. After blocking with 3% H_2O_2 and 10% normal goat serum (CAT# 31872, Thermo Fisher Scientific, USA), sections were incubated with primary antibodies (CD11b, Ly6C, Ki67, and CCR2; Table S7) at 37°C for 1 h, followed by HRP-secondaries and Opal fluorophores. Nuclear counterstaining was performed with 0.5 μg/mL DAPI (CAT# D9542, Sigma-Aldrich, USA) for 5 min. Multispectral images were acquired using the Vectra Polaris system (Akoya, USA) and analyzed with InForm Advanced Image Analysis Software (v2.4).

ELISA experiments

To assess GDF15 levels, the lung of WT mice were collected at 0, 6, 12, 24, 48, and 72 h post ricin challenge. BALF was collected by tracheal catheterization with 0.8 mL PBS. After centrifugation at 3000×g for 5 min at room temperature, supernatants were collected and stored at -80 ℃ for further use. GDF15 levels were measured using ELISA Kit (CAT# SU-BN21657, CZKEVEI, China).

Statistical analysis

Statistical analyses were performed by GraphPad Prism (v9.0). All summary data were expressed as mean ± SD. The exact sample size (n) for each experiment was specified in the relevant figure legends to represent biological replicates. Normality and homogeneity of variance were verified before analysis. Continuous data were analyzed using unpaired Student's *t*-test for two-group comparison, or one-way ANOVA with Tukey's post hoc test for multi-group comparison. Significance thresholds: *: $P<0.05$, **: $P<0.01$, ***: $P<0.001$; ns, not significant ($P\geq0.05$).

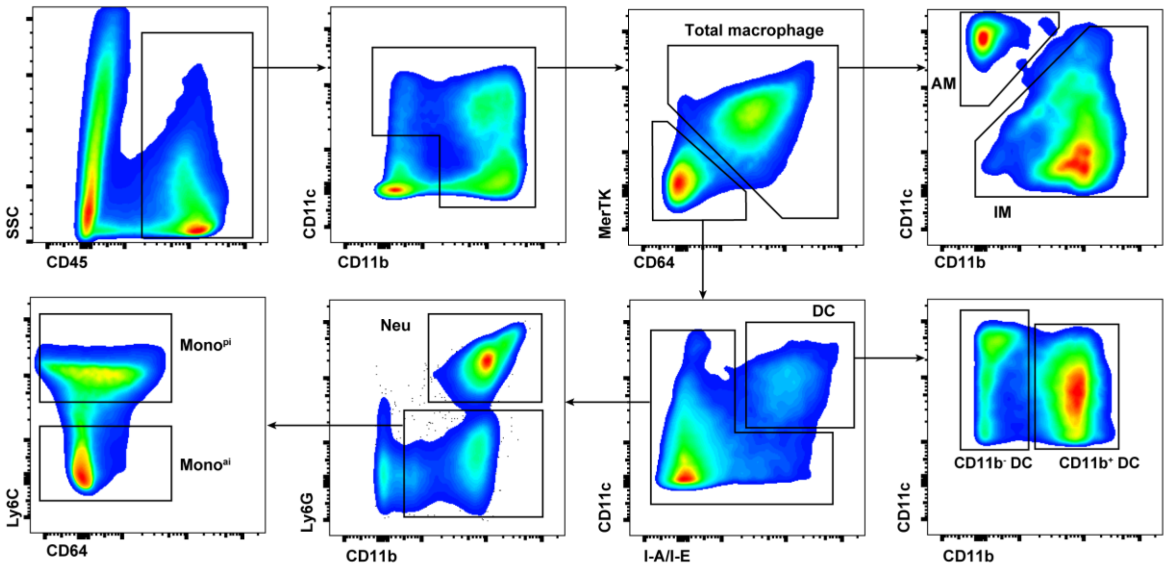

Fig. S1. FCM gating strategy for MNP subpopulations in the DAD lung. $Mono^{pi}$: $CD11b^+$ $MerTK^-$ $CD64^-$ $Ly6G^-$ $Ly6C^{hi}$. $Mono^{ai}$: $CD11b^+$ $MerTK^-$ $CD64^-$ $Ly6G^-$ $Ly6C^{lo}$. Neu: $CD45^+$ $CD11b^+$ $Ly6G^+$. AM: $MerTK^+$ $CD64^+$ $CD11b^-$. IM: $MerTK^+$ $CD64^+$ $CD11b^+$. $CD11b^+$ DC: $MerTK^-$ $CD64^-$ $CD11c^+$ $MHCII^+$ $CD11b^+$. $CD11b^-$ DC: $MerTK^-$ $CD64^-$ $CD11c^+$ $MHCII^+$ $CD11b^-$.

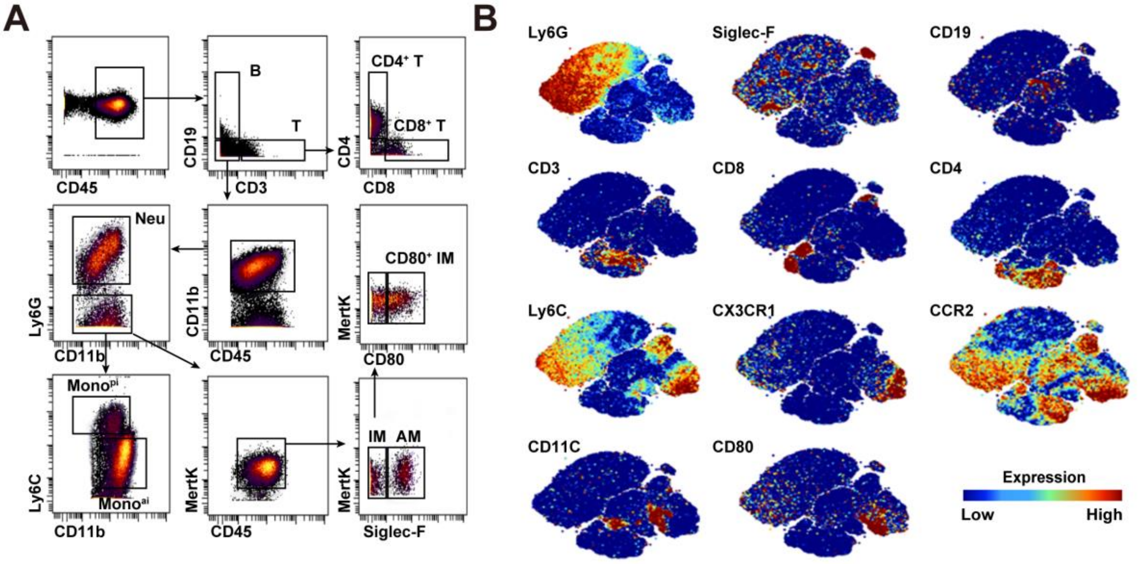

Fig. S2. CyTOF-based identification of major immune cell populations. (A) Gating strategy for major immune cell populations. (B) Expression of markers defining major immune cell populations.

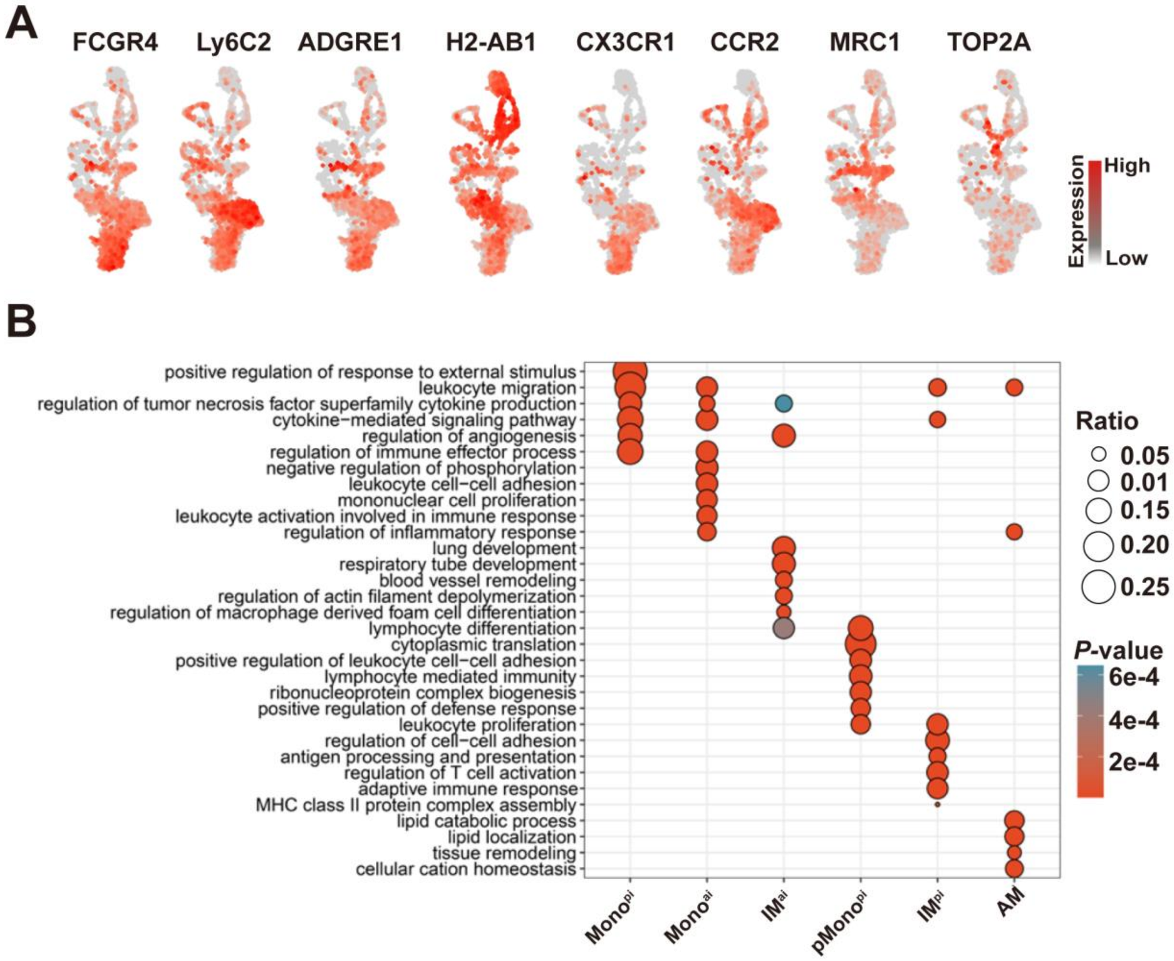

Fig. S3. scRNA-seq-I-based gene expression profiles of MNP subpopulations. (A) Expression pattern of marker genes in various cell clusters. (B) Functional enrichment of biological processes in various cell clusters.

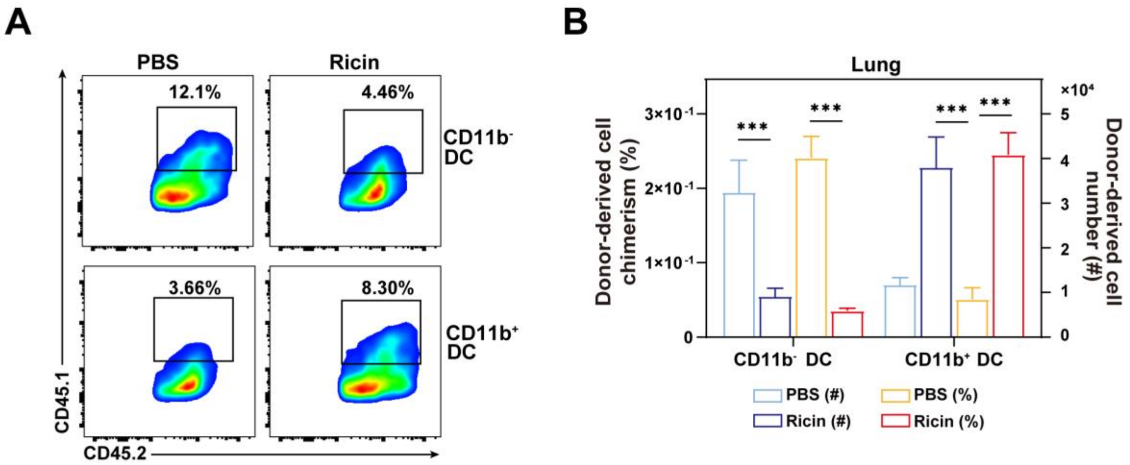

Fig. S4. Adoptive transfer experiments showing circulating Mono as an origin of accumulated $CD11b^+$ DC in the DAD lung. (A) FCM-based proportions of $CD45.1^+$ donor-derived $CD11b^-$ and $CD11b^+$ DC in the $CD45.2^+$ recipient lung. (B) FCM-based quantification of $CD45.1^+$ donor-derived $CD11b^-$ and $CD11b^+$ DC in the $CD45.2^+$ recipient lung. Mice are challenged with PBS or ricin. n=4. Data are presented as mean ± SD. ***: P<0.001, Student's t-test.

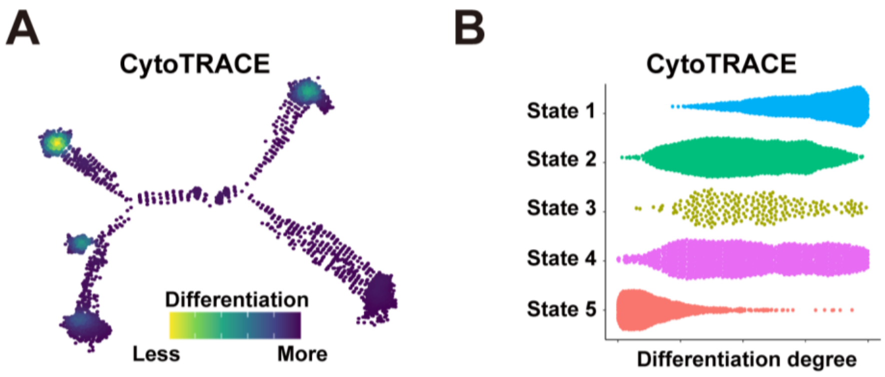

Fig. S5. scRNA-seq-I-based cell differentiation potential analysis in the 5 developmental states. (A) CytoTRACE-based two-dimensional plot of cell differentiation potential. (B) CytoTRACE-based violin plot of cell differentiation potential.

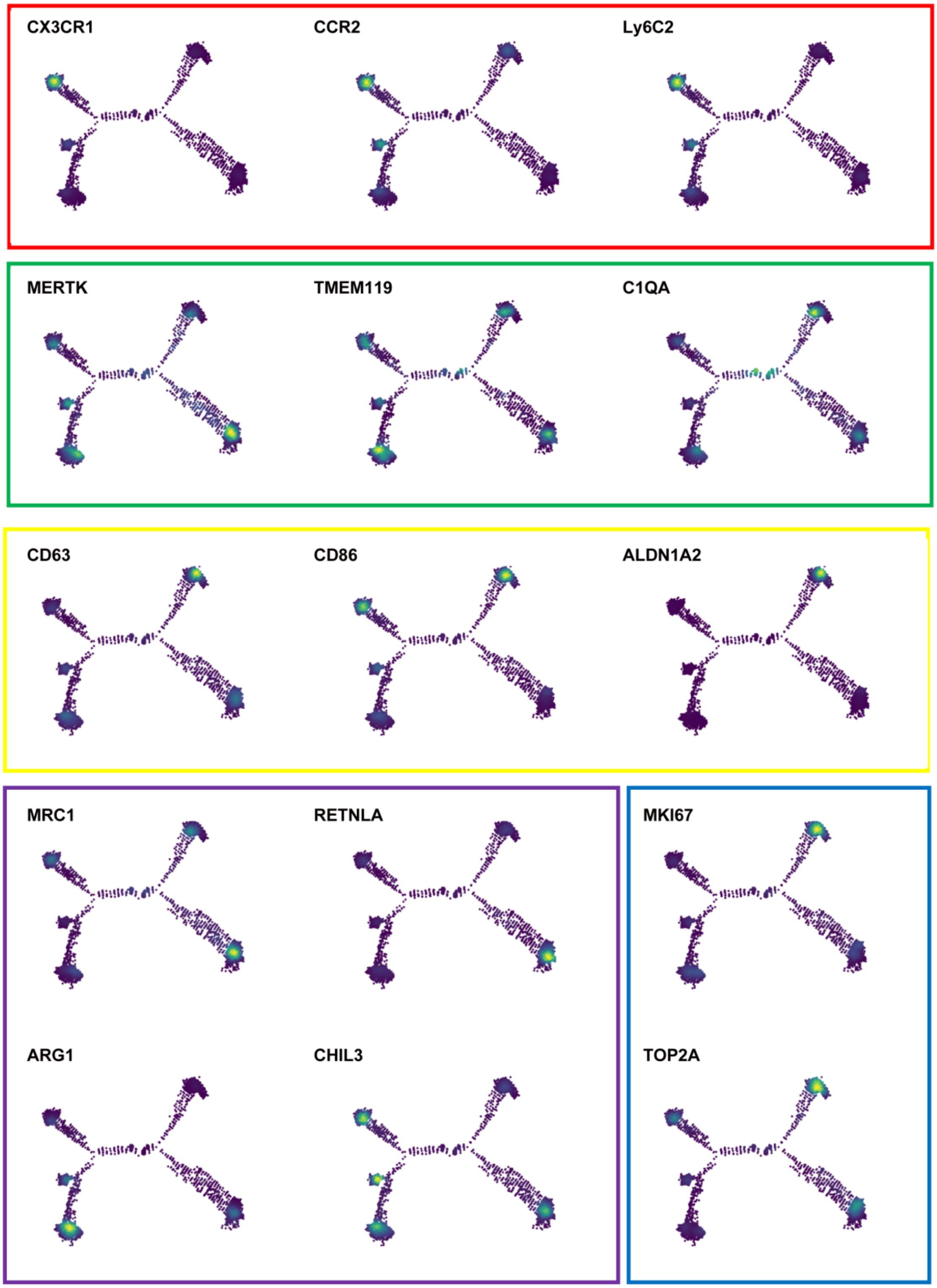

Fig. S6. scRNA-seq-I-based expression pattern of differentiation marker genes in 5 developmental states. Differentiation marker genes: CX3CR1, CCR2, and Ly6C2 (recruited Mono, red); MERTK, TMEM119, and C1QA (IM, green); CD63, CD86, and ALDH1A2 (M1 phenotype, yellow); MRC1, RETNLA, ARG1, and CHIL3 (M2 phenotype, purple); MKI67 and TOP2A (proliferation, blue).

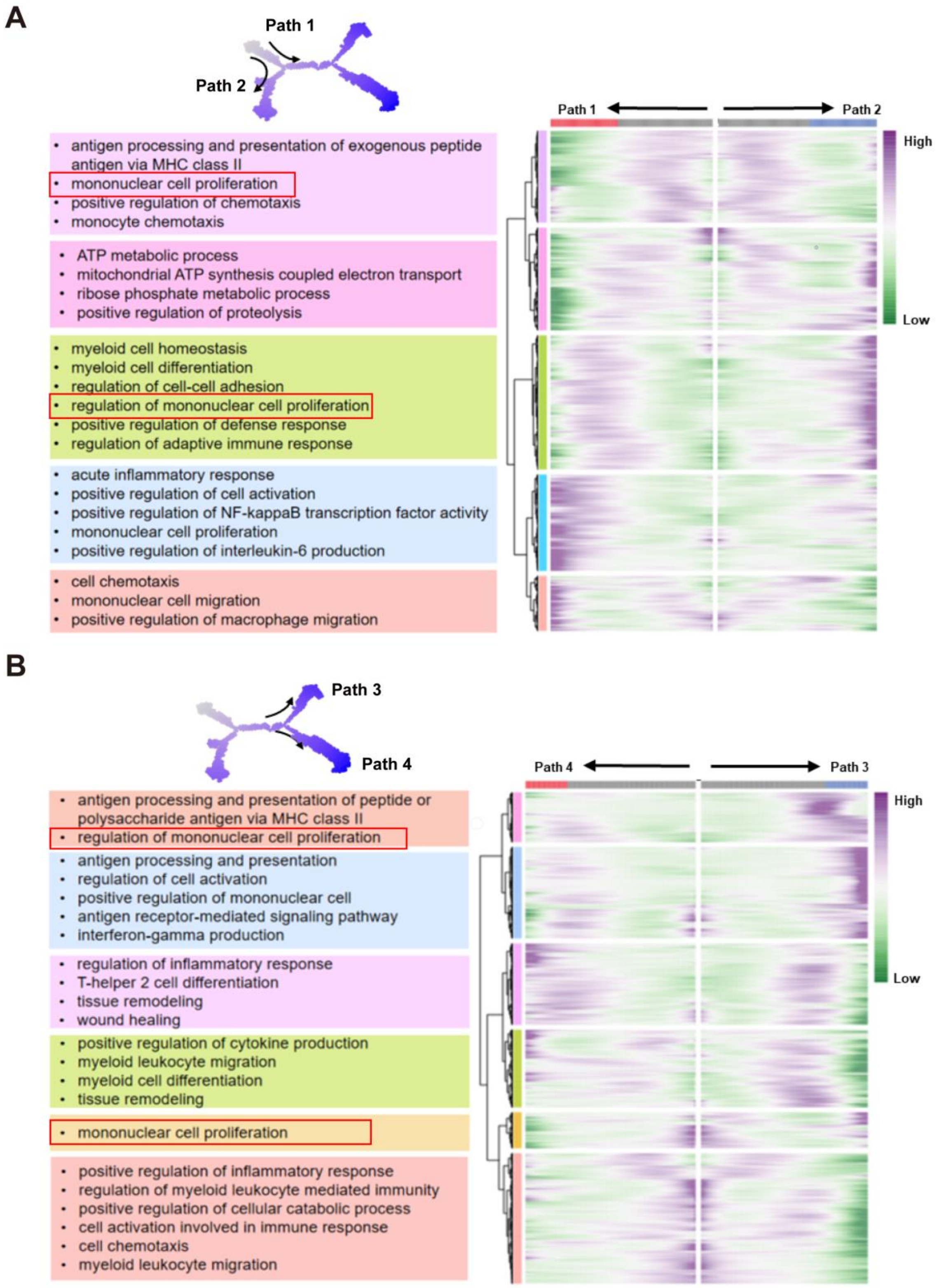

Fig. S7. scRNA-seq-I-based heatmaps of the 5 developmental states of DEGs detected by pseudotime analysis. Heatmap of model-fitted expression values for DEGs and GO enrichment terms (rows, FDR<0.05) along pseudotime in branch 1 (A), and branch 2 (B), as shown in Fig. 4e. Red box indicates items related to 'mononuclear cell proliferation'.

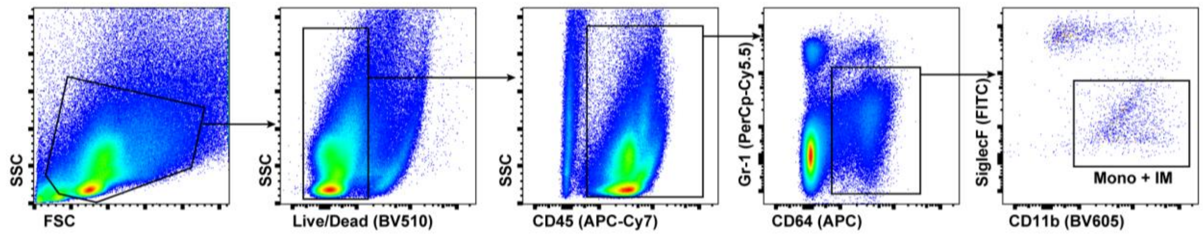

Fig. S8. scRNA-seq-II experiment gating strategy for MNP samples in the DAD lung. After excluding Neu (Gr-1$^+$) and AM (Siglec-F$^+$ CD64$^+$), the remaining cells are gated for Mono, Mono-derived IM, and resident IM with CD11b and CD64. CD11b$^+$ CD64$^+$ cells are sorted for analysis.

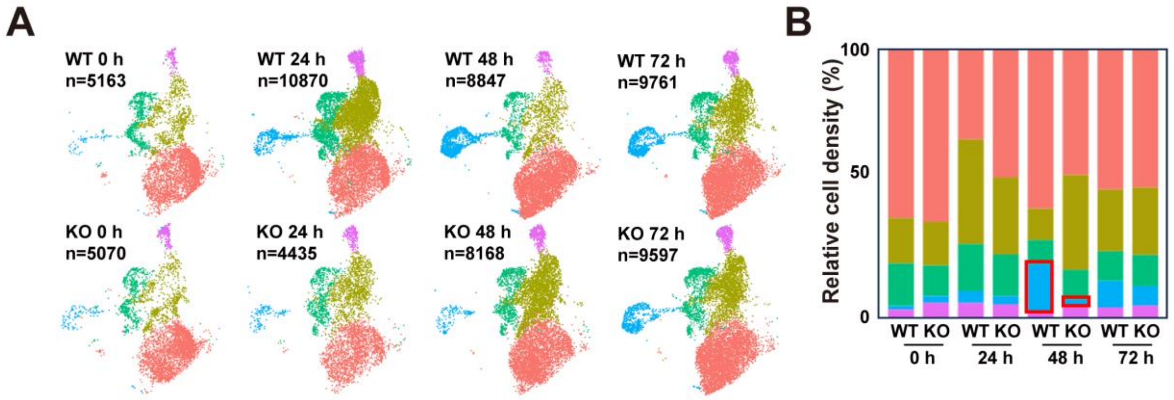

Fig. S9. pMono^{pi} dynamic features in the DAD lung. (A) scRNA-seq-II-based UMAP plots split by DAD timeline and mouse strains (WT and *Gdf15*$^{-/-}$). (B) scRNA-seq-II-based percent of various cell clusters.

Table S1. Staining panel for MNP quantitation.

Antigen_Fluorescent	Clone	Cat#	Company
CD16/32	2.4G2	553141	BD Bioscience
CD45_BUV395	30-F11	564279	BD Bioscience
CD11b_BV605	M1/70	101237	Biolegend
CD64_APC	X54-5/7.1	139305	Biolegend
I-A/I-E(MHC II) _APC-Cy7	M5/114.15.2	107628	Biolegend
MerTK_BV421	2B10C42	25-5751-80	eBioscience
CD11c_PE	N418	117308	Biolegend
Ly6G_BV650	1A8	127641	Biolegend

Table S2. Staining panel for EdU detection.

Antigen_Fluorescent	Clone	Cat#	Company
CD16/32	2.4G2	553141	BD Bioscience
CD11b_FITC	M1/70	101205	Biolegend
CD11c_BV421	N418	117343	Biolegend
Ly6C_BV605	HK1.4	128036	Biolegend
I-A/I-E(MHC II)_BV786	M5/114.15.2	107645	Biolegend
CCR2_PE	SA203G11	150610	Biolegend
SiglecF_PE-Cy7	S17007L	155528	Biolegend
CD45_BUV395	30-F11	564279	BD Bioscience
CD3_PerCP-Cy5.5	17A2	100218	Biolegend
NK1.1_PerCP-Cy5.5	S17016D	156525	Biolegend
CD19_Percp-Cy5.5	6D5	115534	Biolegend
Ly6G_Percp-Cy5.5	1A8	127616	Biolegend
Click-iT Plus Edu Alexa Fluor 647		C10634	Thermo Fisher Scientific
Zombie NIR Fixable Viability Kit		423105	Biolegend

Table S3. Staining panel for CyTOF.

Metal-labeled Antibodies	Clone	Cat#	Company
X89Y_CD45	30-F11	3089005B	Fluidigm
X110Cd_CD39	5F2	135702	Biolegend
X141Pr_Siglec-F	S17007L	155512	Biolegend
X142Ce_EQ2_TNFα	MP6-XT22	506302	Biolegend
X143Nd_CD11b	M1/70	3143015B	Fluidigm
X144Nd_CCR2	475301R	MAB55381R	Novus
X145Nd_CD69	H1.2F3	104533	Biolegend
X146Nd_CD206	C068C2	141702	Biolegend
X148Sm_CD4	GK1.5	100402	Biolegend
X149Sm_IL-6	MP5-20F3	ab259341	Biolegend
X150Sm_MERTK	2B10C42	151502	Biolegend
X151Eu_EQ3_CD68	KP1	NB100-683	Novus
X152Gd_CD3	145-2C11	100345	Biolegend
X153Eu_EQ4_TGFβ	1C5H11	ab166705	Abcam
X154Gd_KI67	11F6	151202	Biolegend
X155Gd_F4/80	BM8	123143	Biolegend
X156Gd_IL-10	1B1.3a	505012	Biolegend
X158Gd_CD19	1D3/CD19	115597	Biolegend
X159Tb_CD73	TY/11.8	127202	Biolegend
X160Dy_CCL8	A16070K	536902	Biolegend
X161Dy_INOS	CXNFT	3161011B	Fluidigm
X162Dy_TIM3	RMT3-23	3162029B	Fluidigm
X163Dy_Ly6C	HK1.4	128039	Biolegend
X164Dy_CX3CR1	SA011F11	3164023B	Fluidigm
X165Ho_EQ5_CD115	AFS98	135521	Biolegend
X166Er_IL-4	11B11	3166003B	Fluidigm
X167Er_CD163	EPR19518	AB213612	Abcam
X168Er_CD8	53-6.7	100755	Biolegend
X169Tm_CD205	NLDC-145	138202	Biolegend
X170Yb_IL-1b	B122	503502	Biolegend
X171Yb_CD80	16-10A1	3171008B	Fluidigm
X172Yb_CD64	X54-5/7.1	139301	Biolegend
X173Yb_VEGFR	89B3A5	121902	Biolegend
X174Yb_Ly6G	RB6-8C5	108449	Biolegend
X175Lu_EQ6_IA-IE	M5/114.15.2	107616	Biolegend
X176Lu_EQ7_PDL1	10F.9G2	124302	Biolegend
X209Bi_CD11c	N418	3209005B	Fluidigm

Table S4. Staining panel for cell sorted in scRNA-seq-I.

Antigen_Fluorescent	Clone	Cat#	Company
CD16/32	2.4G2	553141	BD Bioscience
CD45_Pacific Blue	30-F11	103125	Biolegend
CD11c_PE	N418	117308	Biolegend
CD64_APC	X54-5/7.1	139305	Biolegend
Ly6C_PerCP-Cy5.5	HK1.4	128011	Biolegend
Zombie Aqua Fixable Viability		564406	Biolegend

Table S5. Staining panel for cell sorting in scRNA-seq-II.

Antigen_Fluorescent	Clone	Cat#	Company
CD16/32	2.4G2	553141	BD Bioscience
CD45_BUV395	30-F11	564279	Biolegend
CD64_APC	X54-5/7.1	139305	Biolegend
CD11b_BV605	M1/70	101257	Biolegend
Siglec-F_FITC	S17007L	155503	Biolegend
Gr-1_PerCP-Cy5.5	RB6-8C5	108427	Biolegend
Zombie Aqua Fixable Viability		564406	Biolegend

Table S6. Staining panel for parabiosis and adoptive transfer.

Antigen_Fluorescent	Clone	Cat#	Company
CD16/32	2.4G2	553141	BD Bioscience
CD45_BUV395	30-F11	564279	BD Bioscience
F4/80_FITC	BM8	MAC497FT	Bio-Rad
CD11b_BV605	M1/70	101237	Biolegend
CD64_APC	X54-5/7.1	139305	Biolegend
I-A/I-E(MHC II)_APC-Cy7	M5/114.15.2	107628	Biolegend
MerTK_BV421	ZB10C42	25-5751-80	eBioscience
CD11c_PE	N418	117308	Biolegend
Ly6G_BV650	1A8	127641	Biolegend
Ly6C_PerCP-Cy5.5	HK1.4	128011	Biolegend
CD45.1_PE-Cy7	A20	560578	BD Bioscience
CD45.2_AF700	104	109821	Biolegend
Zombie Aqu Fixable Viability		564406	Biolegend

Table S7. Staining panel for mIHC.

Antigen	Fluorescent	Cat#	Company
CD11b	Opal480	Ab133357	Abcam
Ly6C	Opal570	AB15627	Abcam
Ki67	Opal520	AB16667	Abcam
CCR2	Opal690	AB273050	Abcam

References

Bergen V, Lange M, Peidli S, *et al.* Generalizing RNA velocity to transient cell states through dynamical modeling. *Nat Biotechnol* 2020; **38**: 1408-1414.

Butler A, Hoffman P, Smibert P, *et al.* Integrating single-cell transcriptomic data across different conditions, technologies, and species. *Nat Biotechnol* 2018; **36**: 411-420.

Dai Y, Xu A, Li J, *et al.* CytoTree: an R/Bioconductor package for analysis and visualization of flow and mass cytometry data. *BMC Bioinformatics* 2021; **22**: 138.

Kamran P, Sereti KI, Zhao P, *et al.* Parabiosis in mice: a detailed protocol. *J Vis Exp* 2013; **80**: 50556.

Langfelder P, Horvath S. WGCNA: an R package for weighted correlation network analysis. *BMC Bioinformatics* 2008; **9**: 559.

Qiu P, Simonds EF, Bendall SC, *et al.* Extracting a cellular hierarchy from high-dimensional cytometry data with SPADE. *Nat Biotechnol* 2011; **29**: 886-891.

Qiu X, Hill A, Packer J, *et al.* Single-cell mRNA quantification and differential analysis with Census. *Nat Methods* 2017a; **14**: 309-315.

Qiu X, Mao Q, Tang Y, *et al.* Reversed graph embedding resolves complex single-cell trajectories. *Nat Methods* 2017b; **14**: 979-982.

Su D, Jiao Z, Li S, *et al.* Spatiotemporal single-cell transcriptomic profiling reveals inflammatory cell states in a mouse model of diffuse alveolar damage. *Exploration* 2023; **3**: 20220171.

Van Gassen S, Callebaut B, Van Helden MJ, *et al.* FlowSOM: Using self-organizing maps for visualization and interpretation of cytometry data. *Cytometry A* 2015; **87**: 636-645.

Wu T, Hu E, Xu S, *et al.* clusterProfiler 4.0: A universal enrichment tool for interpreting omics data. *Innovation (Camb)* 2021; **2**: 100141.
